# Machine Learning Algorithms Identify Clinical Subtypes and Cancer in Anti-TIF1γ+ Myositis: A Longitudinal Study of 87 Patients

**DOI:** 10.3389/fimmu.2022.802499

**Published:** 2022-02-14

**Authors:** Lijuan Zhao, Shuoshan Xie, Bin Zhou, Chuyu Shen, Liya Li, Weiwei Pi, Zhen Gong, Jing Zhao, Qi Peng, Junyu Zhou, Jiaqi Peng, Yan Zhou, Lingxiao Zou, Liang Song, Honglin Zhu, Hui Luo

**Affiliations:** ^1^ Department of Rheumatology, Xiangya Hospital of Central South University, Changsha, China; ^2^ National Clinical Research Center for Geriatric Disorders, Xiangya Hospital of Central South University, Changsha, China; ^3^ Provincial Clinical Research Center for Rheumatic and Immunologic Diseases, Xiangya Hospital of Central South University, Changsha, China; ^4^ Department of Nephrology, Hunan Provincial People’s Hospital and The First Affiliated Hospital of Hunan Normal University, Changsha, China; ^5^ Department of Nephrology, The Affiliated Hospital of Qingdao University, Qingdao, China; ^6^ Department of Rheumatology, The First Affiliated Hospital, Sun Yat-sen University, Guangzhou, China; ^7^ Department of Rheumatology, The Third Xiangya Hospital of Central South University, Changsha, China; ^8^ Department of Oncology, The First People’s Hospital of Changde City, Changde, China; ^9^ Department of Rheumatology, Yiyang Central Hospital, Yiyang, China; ^10^ Department of Rheumatology, The First Affiliated Hospital of Jishou University, Jishou, China; ^11^ Department of Rheumatology, Yueyang People’s Hospital, Yueyang, China; ^12^ Huaihua No.1 People’s Hospital Affiliated to Nanhua University, Huaihua, China; ^13^ Department of Obstetrics and Gynecology, Zhuzhou Central Hospital, Zhuzhou, China; ^14^ Department of Obstetrics and Gynecology, The Third Xiangya Hospital of Central South University, Changsha, China

**Keywords:** myositis, anti-TIF1γ antibody, subtypes, prediction, machine learning algorithms, cancer

## Abstract

**Background:**

Anti-TIF1γ antibodies are a class of myositis-specific antibodies (MSAs) and are closely associated with adult cancer-associated myositis (CAM). The heterogeneity in anti-TIF1γ+ myositis is poorly explored, and whether anti-TIF1γ+ patients will develop cancer or not is unknown at their first diagnosis. Here, we aimed to explore the subtypes of anti-TIF1γ+ myositis and construct machine learning classifiers to predict cancer in anti-TIF1γ+ patients based on clinical features.

**Methods:**

A cohort of 87 anti-TIF1γ+ patients were enrolled and followed up in Xiangya Hospital from June 2017 to June 2021. Sankey diagrams indicating temporal relationships between anti-TIF1γ+ myositis and cancer were plotted. Elastic net and random forest were used to select and rank the most important variables. Multidimensional scaling (MDS) plot and hierarchical cluster analysis were performed to identify subtypes of anti-TIF1γ+ myositis. The clinical characteristics were compared among subtypes of anti-TIF1γ+ patients. Machine learning classifiers were constructed to predict cancer in anti-TIF1γ+ myositis, the accuracy of which was evaluated by receiver operating characteristic (ROC) curves.

**Results:**

Forty-seven (54.0%) anti-TIF1γ+ patients had cancer, 78.7% of which were diagnosed within 0.5 years of the myositis diagnosis. Fourteen variables contributing most to distinguishing cancer and non-cancer were selected and used for the calculation of the similarities (proximities) of samples and the construction of machine learning classifiers. The top 10 were disease duration, percentage of lymphocytes (L%), percentage of neutrophils (N%), neutrophil-to-lymphocyte ratio (NLR), sex, C-reactive protein (CRP), shawl sign, arthritis/arthralgia, V-neck sign, and anti-PM-Scl75 antibodies. Anti-TIF1γ+ myositis patients can be clearly separated into three clinical subtypes, which correspond to patients with low, intermediate, and high cancer risk, respectively. Machine learning classifiers [random forest, support vector machines (SVM), extreme gradient boosting (XGBoost), elastic net, and decision tree] had good predictions for cancer in anti-TIF1γ+ myositis patients. In particular, the prediction accuracy of random forest was >90%, and decision tree highlighted disease duration, NLR, and CRP as critical clinical parameters for recognizing cancer patients.

**Conclusion:**

Anti-TIF1γ+ myositis can be separated into three distinct subtypes with low, intermediate, and high risk of cancer. Machine learning classifiers constructed with clinical characteristics have favorable performance in predicting cancer in anti-TIF1γ+ myositis, which can help physicians in choosing appropriate cancer screening programs.

## Introduction

Idiopathic inflammatory myopathies (IIMs, collectively called myositis) are a group of highly heterogeneous systemic autoimmune diseases. IIMs have five main subgroups—dermatomyositis (DM), polymyositis (PM), immune-mediated necrotizing myopathy (IMNM), sporadic inclusion body myositis (sIBM), and overlap myositis (including antisynthetase syndrome) (1, 2). An increased cancer risk in adult myositis patients has been observed in numerous large population studies, and the incidences vary from 6.7% to 32.0% (3–5). Most cancers are diagnosed within 3 years of myositis diagnosis, and the time span of cancer-associated myositis (CAM) is accordingly defined (4, 6–8). DM has a stronger association with cancer than other IIM subgroups, and the cancer types are influenced by the geographical and ethnic backgrounds of myositis patients (9).

Autoantibodies are important biomarkers in IIMs. Myositis-specific autoantibodies (MSAs) are specific to IIMs, while myositis-associated autoantibodies (MAAs) are associated with myositis overlap syndromes. MSAs are closely correlated with distinct disease phenotypes; for example, anti-MDA5 antibodies are associated with clinically amyopathic DM and rapidly progressive interstitial lung disease (RP-ILD) (10).

Transcriptional intermediary factor 1γ (TIF1γ) is a protein belonging to the tripartite motif (TRIM) superfamily that plays diverse roles in transcriptional elongation, DNA repair, cell differentiation, mitosis, and embryonic development. Anti-TIF1γ antibodies were first referred to as anti-p155 in the serum of myositis patients, which immunoprecipitated a 155-kDa nuclear protein (11, 12). Anti-TIF1γ antibodies are found in 7% of adult IIMs and are considered as one of the MSAs present in DM (10, 13, 14). Many studies have reported a strong correlation between anti-TIF1γ antibodies and malignancies. Approximately 50% of anti-TIF1γ+ adult myositis patients are diagnosed with cancers within 3 years (12, 15). However, even if the high risk of cancer in anti-TIF1γ+ myositis is known, physicians still cannot exactly predict the anti-TIF1γ+ myositis patients developing cancer or not at the first diagnosis. More extensive cancer screening is needed for the anti-TIF1γ+ patients who will develop cancer, while less effort is needed for those who will not. Thus, earlier identification of anti-TIF1γ+ patients with probable cancers will be clinically important.

In this study, we analyzed 44 clinical characteristics at baseline of 87 anti-TIF1γ+ adult myositis patients and their outcome of cancer during the follow-up time. Fourteen variables most important for distinguishing cancer and non-cancer were selected. Then, multidimensional scaling (MDS) plot and hierarchical cluster analysis divided anti-TIF1γ+ myositis into three clusters based on sample similarities calculated with these variables, which corresponded to patients with low, intermediate, and high cancer risk. Distinct clinical characteristics were found among clusters. In addition, machine learning classifiers for cancer were constructed and verified with excellent performances. Overall, our study highlighted a new strategy to manage anti-TIF1γ+ patients with machine learning algorithms to stratify and predict their cancer risk at their first visit at hospitals.

## Materials and Methods

### Study Design and Participants

This was a longitudinal study conducted at Xiangya Hospital in Hunan Province, south-central China, from June 2017 to June 2021. The inclusion criteria were a clinical diagnosis of myositis in adult patients according to either the 1975 Bohan/Peter (16) or 2017 EULAR/ACR (1) classification for IIMs, with serological positivity for anti-TIF1γ antibodies, and follow-up for three years from the diagnosis of myositis unless cancer was diagnosed earlier. The exclusion criteria were patients who withdrew or failed to finish the follow-up. Myositis overlapping with other connective tissue diseases, such as systemic lupus erythematosus (SLE) and Sjogren’s syndrome (SS), was not excluded, because overlap myositis is also recognized as one main subtype of IIMs (2). The antibody repertoire of myositis was detected with serum using commercially available kits (Euroimmun, Germany, and KingMed, China). A flow diagram of the study protocol is shown in **Supplementary File 1**. This study was approved by the Ethics Committee of the Institutional Review Board at Xiangya Hospital (#201212074), and written informed consent was obtained from all patients.

### Data Collection

Medical records of patients at the time of enrollment before treatment were reviewed, and data on 44 clinical variables (**Supplementary File 2**) were collected with no missing data. In addition, the cancer categories and time interval from myositis diagnosis to cancer diagnosis were also obtained. In our study, CAM was defined as cancers occurring within 3 years of the myositis diagnosis (before or after) (17).

### Variable Selection, Clustering Analysis, and Construction of Machine Learning Classifiers

Machine learning algorithms were performed in R or Python. Elastic net was used to select the most important clinical variables out of all 44 clinical variables for the classification of anti-TIF1γ+ myositis with cancer or not. The importance of selected variables was evaluated by random forest with mean decrease Gini. The similarities (random forest proximities) of samples were calculated with the selected variables, based on which multidimensional scaling (MDS) plot and hierarchical cluster analysis were used to cluster and visualize these samples. Five machine learning classifiers [random forest, support vector machines (SVM), extreme gradient boosting (XGBoost), elastic net, and decision tree] were also constructed with these selected clinical variables to predict cancer probability of anti-TIF1γ+ patients. Stratified 10-fold cross-validation was performed to estimate the accuracy of each model and optimize the models with hyperparameter tuning. Eighty-seven patients were split into a training set containing 70% of the observations and a test set containing the remaining 30%. The training set was used to build the classificatory models, and the testing set was used to evaluate the accuracy of the model. All steps were performed using Python V.3.9.1, scikit-learn V.0.24.2, NumPy V.1.19.5, and pandas V.1.2.1. ROC (receiver operating characteristic) curves were drawn with Matplotlib V.3.3.3.

### Statistical Analysis

The statistical analysis of comparisons among anti-TIF1γ+ myositis patients in the three clusters was performed in SPSS v.22 or GraphPad 8.0, and *p <*0.05 was considered statistically significant. Quantitative data were described as the means (standard deviations) or medians (interquartile ranges) according to data distribution and homoscedasticity. Qualitative data were described as frequencies (percentages). Accordingly, one-way ANOVA or Kruskal–Wallis tests (adjusted with Dunnett T3 or Dunn’s test) and Pearson chi-square test or Fisher’s exact test (adjusted with Bonferroni test) were performed to compare multiple groups. The heatmap was plotted by ggplot2 in R.

## Results

### Characteristics of 87 Anti-TIF1γ+ Patients and Their Temporal Relationship Between Myositis and Cancer

Anti-TIF1γ+ myositis patients enrolled in our study were mainly female (64.4%), with an average age of 57 years and a median disease duration of 6 months. Their most common manifestation was typical DM rashes (95.4%), followed by proximal weakness (56.3%), ILD (17.2%), and arthritis/arthralgia (11.5%). Two patients (2.3%) were complicated with another connective tissue disease—Sjogren’s syndrome.

**Figure 1:**
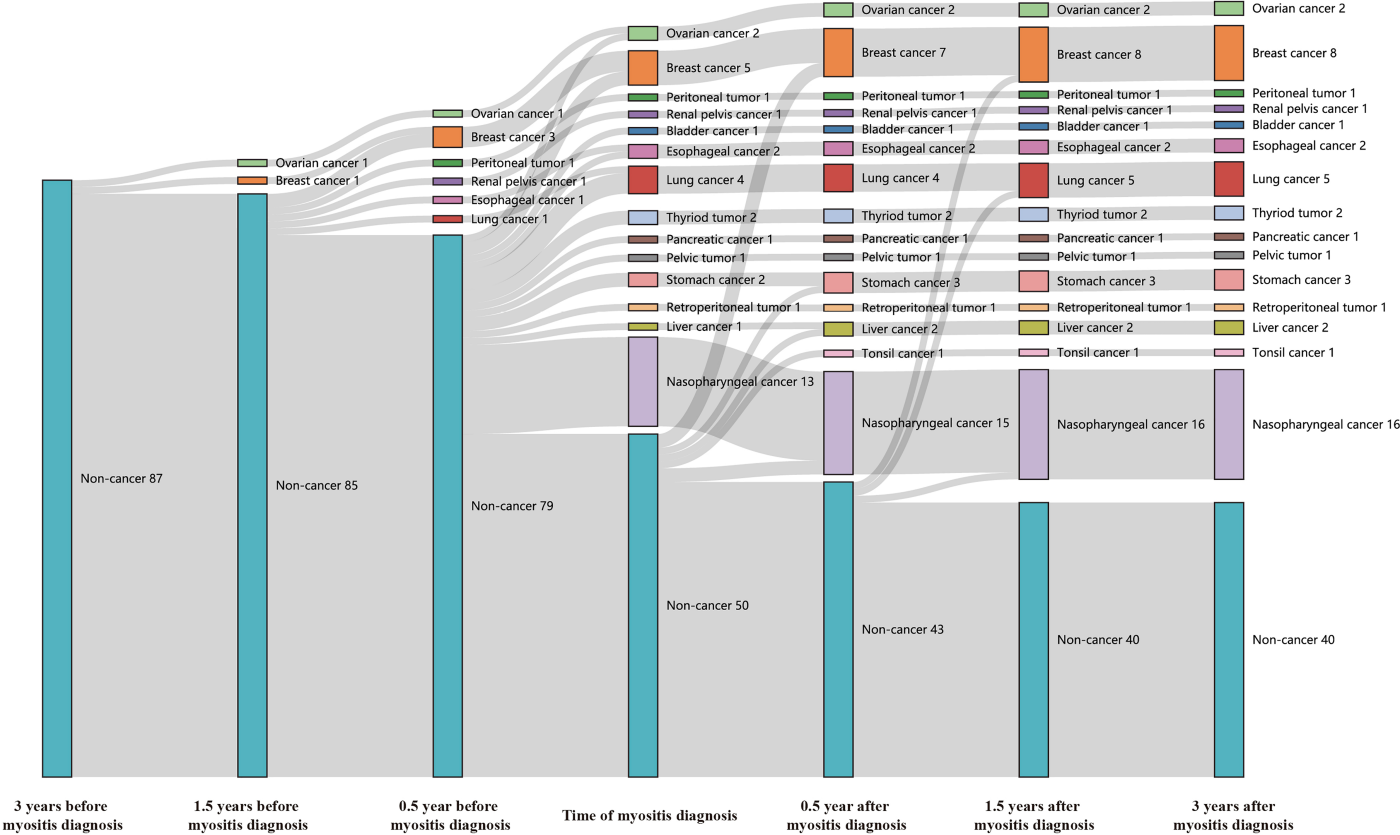
The Sankey diagram showed temporal relationships between the diagnosis of myositis and cancer in 87 anti-TIF1γ+ myositis patients. Seven time points, including 0.5, 1.5, and 3 years before or after myositis diagnosis and the time of the myositis diagnosis, were analyzed.

Forty-seven (54.0%) of our anti-TIF1γ+ patients in total met the diagnostic criteria of CAM. Their temporal relationships between the diagnosis of myositis and cancer are shown in **Figure 1**. None of the anti-TIF1γ+ patients had cancer until 1.5 years before the myositis diagnosis when two female patients were diagnosed with breast and ovarian carcinomas. From 0.5 to 1.5 years before the myositis diagnosis, six cases of cancer were diagnosed, consisting of five female patients and one male patient. Within 0.5 years before and after the myositis diagnosis, 36 anti-TIF1γ+ patients were diagnosed with cancer, and 72.2% of them had the diagnosis of cancer and myositis simultaneously. Nasopharyngeal carcinoma was the most frequent cancer type, and all cases were developed in males. During the period of 0.5 to 1.5 years after the myositis diagnosis, only three more patients (two male and one female) were reported to have cancer. No cancer was reported in the remaining follow-up time. Overall, cancer was most frequently diagnosed within 0.5 years before and after the myositis diagnosis, especially at the time of myositis diagnosis. A total of 45.0% (9/20) female and 7.4% (2/27) male patients had an earlier diagnosis of cancer before myositis developed. Nasopharyngeal carcinoma, breast cancer, and lung cancer were the top 3 tumor categories with the highest incidence.

### Subtypes in Anti-TIF1γ+ Myositis Patients

**Figure 2:**
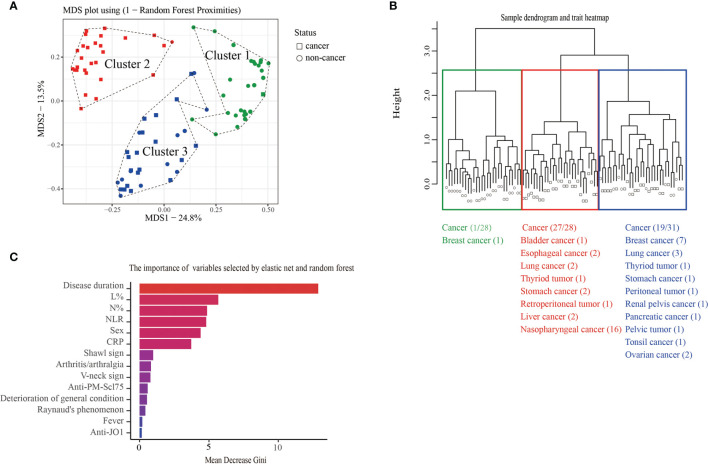
**(A)** The MDS plot and **(B)** hierarchical cluster analysis of 87 anti-TIF1γ+ myositis patients based on random forest proximities that were calculated by 14 clinical variables showed three distinct clusters and the distribution of cancer in each cluster. **(C)** The bar plot represents the importance of clinical variables evaluated by random forest.

Anti-TIF1γ+ patients exhibit differences in whether complicated with cancer or not. Using the elastic net and random forest method, 14 variables that contribute most to discriminating between cancer and non-cancer were selected. To identify the heterogeneities among anti-TIF1γ+ myositis, the similarities (random forest proximities) of patients were calculated by these 14 variables. As shown in the MDS plot and hierarchical clustering tree (**Figures 2A, B** **)**, the 87 anti-TIF1γ+ patients can be separated into three distinct clusters. The patient numbers in clusters 1, 2, and 3 were 28 (32.2%), 28 (32.2%), and 31(35.6%), respectively. Twenty-seven (96.4%) patients in cluster 2 developed cancer, which was almost the opposite of cluster 1 with only one (3.6%) cancer patient. Cluster 3 had 19 (61.3%) patients with cancer and were intermediate between clusters 1 and 2. Therefore, we inferred clusters 1, 2, and 3 corresponded to three stratifications with low, high, and intermediate cancer risk. The importance of the 14 selected clinical variables was ranked with random forest algorithm according to mean decrease Gini (**Figure 2C**). The top 10 important variables were disease duration, percentage of lymphocytes (L%), percentage of neutrophils (N%), neutrophil-to-lymphocyte ratio (NLR), sex, C-reactive protein (CRP), shawl sign, arthritis/arthralgia, V-neck sign, and anti-PM-Scl75 antibodies.

### Comparison of Clinical Characteristics Among the Three Clusters of Anti-TIF1γ+ Myositis

**Figure 3:**
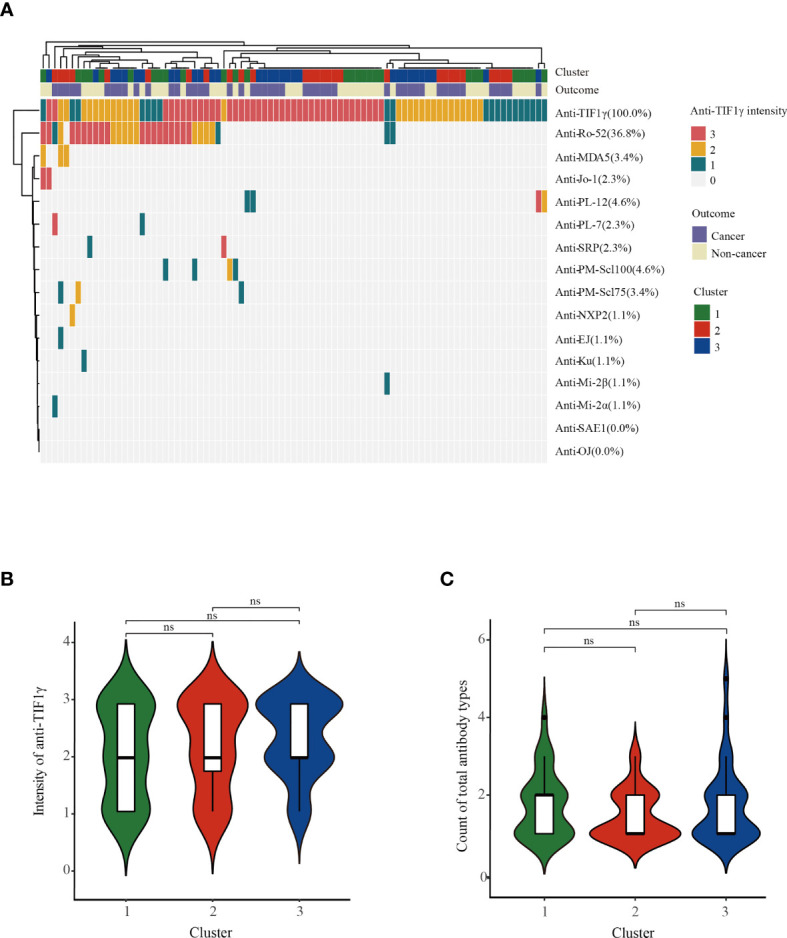
**(A)** The heatmap showed the autoantibody profile of 87 anti-TIF1γ+ myositis patients. **(B)** The comparison of anti-TIF1γ intensities and **(C)** the comparison of count of total antibody types among anti-TIF1γ+ myositis patients in three clusters. n.s. represents no significance.

We then analyzed the clinical characteristics of anti-TIF1γ+ patients in the three clusters (**Table 1**). Significant intergroup differences between cluster 1 (low-risk cancer group) and cluster 2 (high-risk cancer group) were found. For instance, patients in cluster 2 were all male, but most patients in cluster 1 are female (*p* < 0.001). Patients in cluster 2 had an older age (*p* = 0.020); a higher percentage of skin ulcers (*p* = 0.008); a shorter disease duration (*p* < 0.001); higher levels of CRP (*p* = 0.005), lactic dehydrogenase (LDH) (*p* < 0.001), creatine kinase (CK) (*p* = 0.005), N% (*p* = 0.013), and NLR (*p* < 0.001); and a lower level of L% (*p* < 0.001). Clusters 1 and 3 also had significant difference in disease duration (*p* < 0.001), LDH (*p* < 0.001), N% (*p* < 0.001), L% (*p* < 0.001), and NLR (*p* < 0.001). Interestingly, clusters 2 and 3 differed obviously in cancer types: 87.5% patients with breast cancer were in cluster 3 and 100.0% patients with nasopharyngeal carcinoma were in cluster 2 (*p* = 0.005 and *p* < 0.001). Ovarian cancer, peritoneal tumor, renal pelvis cancer, pancreatic cancer, pelvic tumor, and tonsil cancer were all developed in cluster 3. Conversely, bladder cancer, esophageal cancer, retroperitoneal cancer, and liver cancer were all found in cluster 2 (**Figure 2**). The autoantibody profile of myositis in our patients is displayed in **Figure 3A**. A total of 14 (16.1%) anti-TIF1γ+ myositis patients had other MSAs, such as anti-MDA5, anti-Jo1, and anti-PL12. Anti-Ro52 was the most common MAAs. However, there was no difference in the intensities of anti-TIF1γ antibodies or the count of total antibody types among anti-TIF1γ+ myositis patients in the three clusters (**Figures 3B, C** **)**.

**Table 1:** Characteristics of anti-TIF1γ+ myositis patients among three clusters at the time of first visit at our hospital.

	Cluster 1 *n* = 28	Cluster 2 *n* = 28	Cluster 3 *n* = 31	Global *p*_adj_
**Age (years)**	50 ± 18^#^	61 ± 10	58 ± 14	0.018
**Sex**				
Men	3 (10.7%)^#^	28 (100.0%)	0 (0.0%)^&^	<0.001
Women	25 (89.3%)^#^	0 (0.0%)	31 (100.0%)^&^	
**Disease duration (months)**	40.5 (49.5)^#*^	3.0 (6.25)	5.0 (10.0)	<0.001
**General condition/inflammation**				
Fever	1 (3.6%)	2 (7.1%)	1 (3.2%)	0.836
Deterioration of general condition	0 (0.0%)	3 (10.7%)	2 (6.5%)	0.272
ESR (mm/h)	26 (52)	55 (57)	33 (38)	0.127
CRP (mg/L)	2.52 (5.85)^#^	14.25 (17.85)	3.12 (5.00)	0.007
**Skin lesions**				
Heliotrope rash	24 (85.7%)	26 (92.9%)	27 (87.1%)	0.765
Gottron’s sign	12 (42.9%)	16 (57.1%)	16 (51.6%)	0.572
V-neck sign	15 (53.6%)	20 (71.4%)	18 (58.1%)	0.408
Shawl sign	11 (39.3%)	15 (53.6%)	12 (38.7%)	0.451
Holster sign	2 (7.1%)	7 (25.0%)	7 (22.6%)	0.186
Mechanic’s hands	1 (3.6%)	0 (0.0%)	3 (9.7%)	0.319
Raynaud phenomenon	0 (0.0%)	0 (0.0%)	2 (6.5%)	0.326
Skin ulcers	0 (0.0%)^#^	6 (21.4%)	1 (3.2%)	0.008
**Muscular manifestations**				
Proximal weakness	11 (39.3%)	17 (60.7%)	21 (67.7%)	0.076
LDH (U/L)	211.4 (88.9)^#*^	327.5 (209.7)	355.1 (161.0)	<0.001
CK (U/L)	81.1 (161.2)^#^	403.1 (2008.0)	156.0 (355.4)	0.006
**Lung manifestations**				
ILD	4 (14.3%)	5 (17.9%)	6 (19.4%)	0.938
Lung infection	1 (3.6%)	7 (25.0%)	2 (6.5%)	0.049
**Other rheumatologic manifestations**				
Arthritis/arthralgia	4 (14.3%)	3 (10.7%)	3 (9.7%)	0.916
**Blood cells**				
WBC (×10^9^/L)	5.5 (1.6)	6.7 (4.4)	6.8 (2.7)	0.090
Hb (g/L)	124 ± 17^*^	122 ± 15	112 ± 18	0.010
N%	65.4 (14.0)^#*^	72.2 (13.5)	76.7 (13.1)	<0.001
L%	25.3 ± 7.0^#*^	14.5 ± 4.9	15.0 ± 7.3	<0.001
NLR	2.9 (1.9)^#*^	5.1 (3.7)	6.1 (4.6)	<0.001
M%	8.5 (5.2)	9.9 (3.4)	8.0 (3.9)	0.067
**Cancer**	1 (3.6%)^#*^	27 (96.4%)	19 (61.3%)^&^	<0.001

ESR, erythrocyte sedimentation rate; CRP, C-reactive protein; LDH, lactic dehydrogenase; CK, creatine kinase; ILD, interstitial lung disease; WBC, white blood cells; RBC, red blood cells; N%, percentage of neutrophils; L%, percentage of lymphocytes; NLR, neutrophil-to-lymphocyte ratio; M%, percentage of monocytes.

^#^p < 0.05 for comparison between cluster 1 and cluster 2; ^*^p < 0.05 for comparison between cluster 1 and cluster 3; ^&^p < 0.05 for comparison between cluster 2 and cluster 3.

### Machine Learning Classifiers Predicting Cancer in Anti-TIF1γ+ Patients

**Figure 4:**
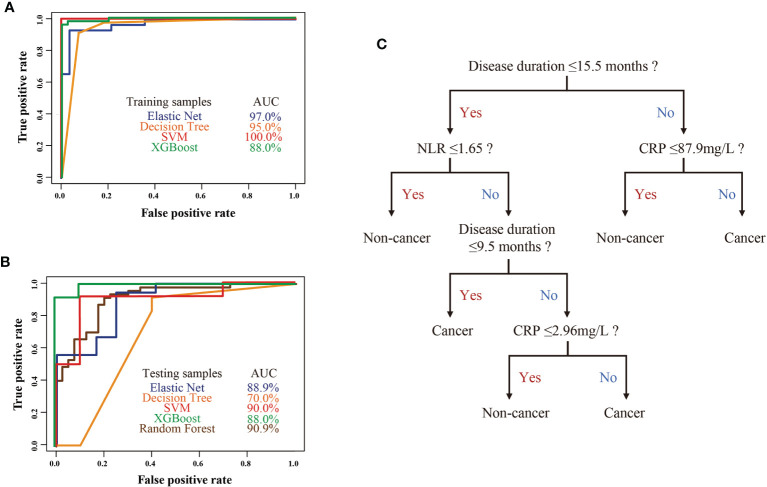
**(A)** The ROC curves of four machine learning models in the anti-TIF1γ+ myositis training samples. **(B)** The ROC curves of four machine learning models (elastic net, decision tree, SVM, XGBoost) in the anti-TIF1γ+ myositis testing samples and random forest model in all patient samples. **(C)** Decision tree model showed disease duration, CRP, and NLR as important clinical features in the prediction of cancer in anti-TIF1γ+ myositis patients.

Early diagnosis of cancer in anti-TIF1γ+ myositis is crucial for improving prognosis, especially for patients with intermediate and high cancer risk in clusters 2 and 3. We constructed machine learning models with the selected 14 most important variables to predict anti-TIF1γ+ myositis with or without cancer. The ROC curves of machine learning models calculated using the training and testing sets are shown in **Figure 4**. The SVM model had an AUC (area under the ROC Curve) of 100.0% in the training set and 90.0% in the test set. The elastic net and XGBoost models also had good performance with AUCs higher than 85.0%. In addition, we trained the random forest model with all 87 samples and the AUC was 90.9%. To conveniently predict the cancer risk of anti-TIF1γ+ myositis patients in clinical practice, we built a decision tree model in the training set (**Figure 4C**). This model included three variables—disease duration, NLR, and CRP. Its AUCs in the training set and test set were 95.0% and 70.0% (**Figures 4A, B** **)**, respectively.

## Discussion

In this study, anti-TIF1γ+ myositis is separated into three different clinical subtypes. Clusters 1, 2, and 3 correspond to patients with low, high, and intermediate risk of cancer, respectively. Anti-TIF1γ+ patients with low cancer risk have distinct clinical characteristics from those with high and intermediate cancer risk, which enables the construction of models to predict cancer in anti-TIF1γ+ patients. Indeed, machine learning classifiers (random forest, SVM, elastic net, decision trees, and XGBoost) showed good classification in discriminating anti-TIF1γ+ patients with cancer or not. In particular, the prediction accuracy of random forest was >90%. Decision tree can conveniently stratify and manage anti-TIF1γ+ myositis patients with disease duration, NLR, and CRP in clinical practice. It is of great value because myositis patients who are positive for anti-TIF1γ antibodies and predicted to develop cancers by our models should undergo more careful and intensive tumor screening than those predicted with low cancer risk.

Fourteen clinical variables were selected for model construction in our study. The top 10 variables ranked by importance were disease duration, L%, N%, NLR, sex, CRP, shawl sign, arthritis/arthralgia, V-neck sign, and anti-PM-Scl75 antibodies. The disease duration (from myositis onset to visit at our hospital) was shorter in the cluster with intermediate and high cancer risks than in the cluster with low cancer risk. This may be related to the more severe disease state of patients in clusters with higher cancer risk, manifested as more patients with deteriorated general condition and increased CK levels. Anti-TIF1γ+ myositis patients in clusters with intermediate and high cancer risk had a higher N% and NLR but a lower L%. Neutrophil dysregulation is pathogenic in myositis, mainly through neutrophil extracellular traps (NETs) and the subset of low-density granulocytes (LDGs) (18). The underlying mechanisms for decreased L% are uncertain and need further exploration. Our anti-TIF1γ+ myositis patients in cluster 2 with the highest risk of cancer were more often males, had higher CRP levels, and more frequent shawl sign and V-neck sign. These clinical characteristics were proven to be related factors for CAM (9, 19–22). For example, a meta-analysis including 69 studies concluded that DM subtype, older age, male gender, dysphagia, cutaneous ulceration, and anti-TIF1γ positivity were associated with increased cancer risk (21). Another large-scale multicenter cohort study found CAM patients had more common older age, heliotrope rash, shawl sign, and V sign (22). In fact, anti-TIF1γ+ myositis patients with cancer account for nearly half of the CAM cases, that is why they exhibit large clinical similarities (15, 17).

There were no differences in anti-TIF1γ intensities (at the time of visit in our hospital) among clusters with different cancer risks. It is still controversial whether the levels of anti-TIF1γ antibodies correlate with the evolution of cancer (23–25). Tests of anti-TIF1γ antibodies at different time points (e.g., after cancer therapy) might be helpful. Although 41 (47.1%) anti-TIF1γ+ patients had more than one type of autoantibody, we found no association between cancer risk and the number of antibody types in our study. All three anti-TIF1γ+ patients with anti-PM-Scl75 antibodies developed cancer in our study. Anti-PM-Scl75 antibodies are usually suggestive of overlapping myositis accompanied by the manifestation of systemic sclerosis (26, 27). The relationship between anti-PM-Scl75 antibodies and CAM is unknown. In another Chinese CAM cohort, a higher positive rate of anti-PM-Scl75 antibodies was also found in cancer patients than in cancer-free patients (4.1% vs. 2.8%) (28). In our study, one anti-TIF1γ+ patient with cancer was positive for another MSA also related to cancer—anti-NXP2 antibody (29, 30). The standardized incidence ratio of anti-TIF1γ antibodies for estimating cancer risk was 17.28, twice that of anti-NXP2 antibodies (17). It is reasonable to believe that anti-TIF1γ antibodies are predominant to link with the presence of cancer in our myositis patient. There are some new potential biomarkers of cancer in myositis: the IgG2 isotype of anti-TIF1γ helps to identify the risk of mortality in anti-TIF1γ+ patients (31); soluble programmed death ligand 1 (sPD-L1), combined with anti-TIF1γ antibodies, yielded greater specificity and positive predictive value in diagnosing cancer, reaching values of 95% and 70%, respectively (32). These indexes show promising values in clinical practice and needed further verification in larger study populations.

Our study showed that 78.7% (37/47) of cancers were diagnosed within 0.5 years of the myositis diagnosis. A time span of 3 years before and after myositis diagnosis is emphasized in the definition of CAM (4, 6–8). In fact, most cancers were diagnosed simultaneously with or during the first year after the diagnosis of myositis according to previous studies (7, 33). Anti-TIF1γ was significantly associated with a shorter time between myositis and cancer onset (34–36). In one study with 10-year follow-up, all the detected malignancy cases in the anti-TIF1γ+ cohort occurred between 3 years prior to and 2.5 years after DM onset, whereas cancers were detected in the following 7.5 years in anti-TIF1γ- patients (34). We also found 23.4% (11/47) of cancers diagnosed before the myositis diagnosis; 81.8% (9/11) of these patients were females in cluster 3. This may be partially explained by the theory of paraneoplastic myositis syndrome, which regards manifestations of the skin and skeletal muscle as consequences of underlying malignancy (37). Nasopharyngeal carcinoma (34.0%), breast cancer (17.0%), and lung cancer (10.6%) were the 3 three cancers with the highest incidence in our cohort. The organs where myositis patients are prone to develop a tumor vary in different studies (8, 38–40). This is influenced mainly by genetic background and ethnicity (9). The finding in our study is comparable with the local general population because most patients enrolled in the study live in southeastern China—an area with a high incidence of nasopharyngeal cancer. Interestingly, the nasopharynx and breast were the most common cancer sites in male and female patients, respectively, emphasizing different screening targets in male and female patients.

This is the first study identifying subtypes and predicting cancer in anti-TIF1γ+ myositis by machine learning algorithms, although several limitations exist. First, compared with anti-MDA5 (30%) and anti-JO1 (19%) antibodies, the positive rate of anti-TIF1γ antibodies (7%) among myositis patients is relatively low (41). Thus, multicenter cooperation and a large sample size are needed for further study. Second, the mechanism by which anti-TIF1γ+ myositis patients develop cancer was not explored in our study. We can predict the cancer risk of anti-TIF1γ+ myositis patients and prompt early discovery but cannot prevent the occurrence of cancer from the pathogenesis of the disease.

In conclusion, anti-TIF1γ+ myositis can be divided into three distinct subtypes based on their clinical characteristics, which are corresponding to patients with low, intermediate, and high cancer risk. Machine learning models showed satisfactory accuracy in the prediction of cancer in anti-TIF1γ+ myositis patients. These findings suggested that the stratified management of anti-TIF1γ+ myositis patients is necessary to avoid excessive cancer screening examinations in patients with low cancer risk and make the best use of targeted cancer screening in patients predicted to develop cancer.

## Data Availability Statement

The raw data supporting the conclusions of this article will be made available by the authors, without undue reservation.

## Ethics Statement

The studies involving human participants were reviewed and approved by the Ethics Committee of the Institutional Review Board at Xiangya Hospital. The patients/participants provided their written informed consent to participate in this study.

## Author Contributions

LZ, HZ, and HL designed the study. SX, BZ, CS, ZG, WP, LL, JiZ, QP, JuZ, JP, YZ, LZ, and LS collected the data. LZ and HZ analyzed the results and wrote the draft. HL revised the manuscript for content. All authors contributed to the article and approved the submission for publication.

## Funding

This work was supported by grants from the National Natural Science Foundation of China (81771765, 81701621) and Hunan Provincial Natural Science Foundation (2019JJ40503).

## Conflict of Interest

The authors declare that the research was conducted in the absence of any commercial or financial relationships that could be construed as a potential conflict of interest.

## Publisher’s Note

All claims expressed in this article are solely those of the authors and do not necessarily represent those of their affiliated organizations, or those of the publisher, the editors and the reviewers. Any product that may be evaluated in this article, or claim that may be made by its manufacturer, is not guaranteed or endorsed by the publisher.
