# Supplementary figures and images for: Machine Learning Algorithms Identify Clinical Subtypes and Cancer in Anti-TIF1γ+ Myositis: A Longitudinal Study of 87 Patients
Source: Front Immunol. 2022 Feb 14;13:802499. doi: 10.3389/fimmu.2022.802499 (PMC8883045; doi:10.3389/fimmu.2022.802499)

Supplementary file 1

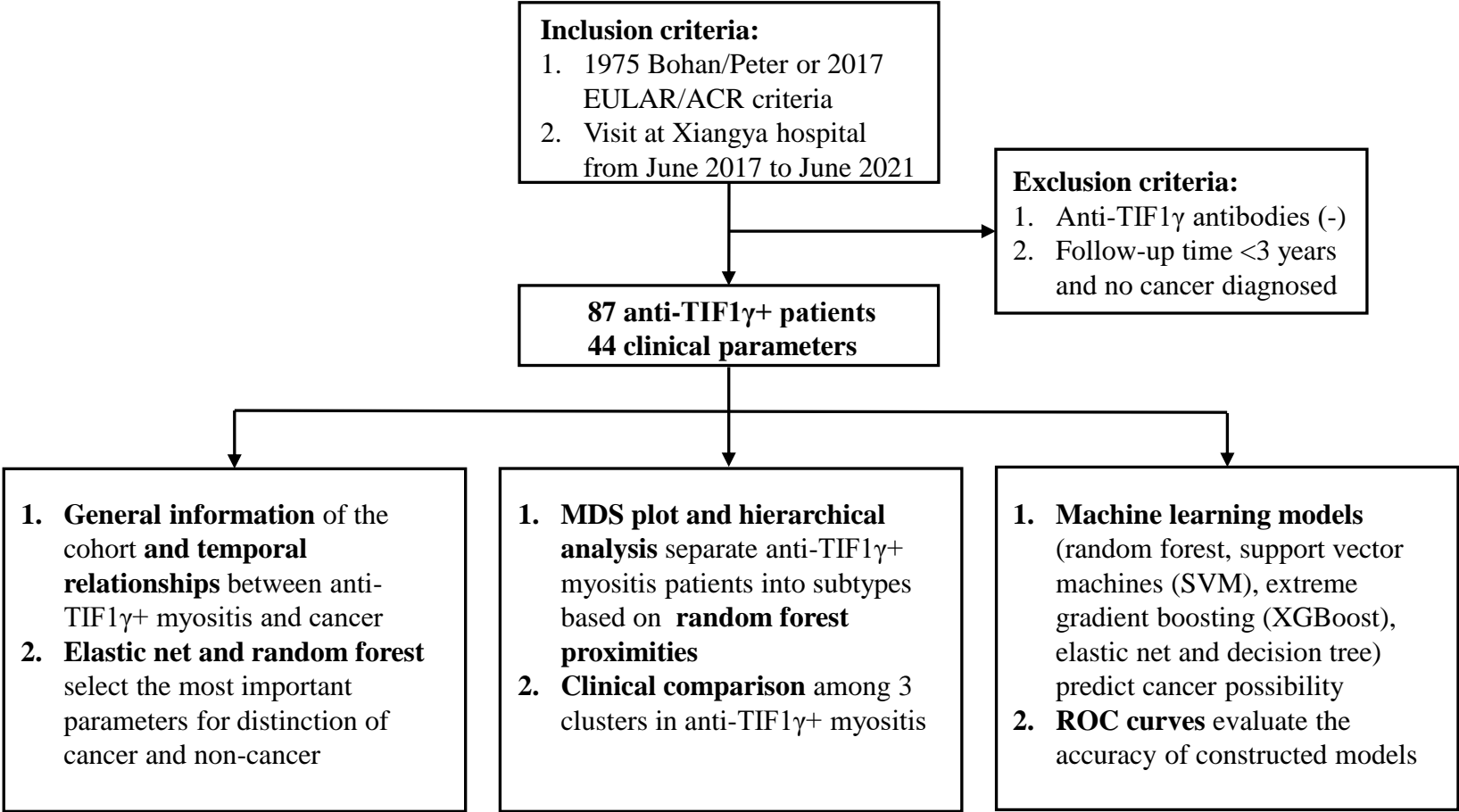

Supplement: Supplementary file 1 [file DataSheet_1.pdf]
