# Supplementary material for: Machine Learning Algorithms Identify Clinical Subtypes and Cancer in Anti-TIF1γ+ Myositis: A Longitudinal Study of 87 Patients
Source: Front Immunol. 2022 Feb 14;13:802499. doi: 10.3389/fimmu.2022.802499 (PMC8883045; doi:10.3389/fimmu.2022.802499)
Supplement: Supplementary file 2 [file DataSheet_2.docx]

**Supplementary file 2**

**44 clinical parameters used in analysis**

sex, age, disease duration, intensity of anti-Mi-2α, intensity of anti-Mi-2β, intensity of anti-TIF1γ, intensity of anti-MDA5, intensity of anti-NXP2, intensity of anti-SAE1, intensity of anti-Ku, intensity of anti-PM-Scl100, intensity of anti-PM-Scl75, intensity of anti-Jo1, intensity of anti-SRP, intensity of anti-PL7, intensity of anti-PL12, intensity of anti-EJ, intensity of anti-OJ, intensity of anti-Ro52, presence of other MSAs beside anti-TIF1γ, deterioration of general condition, fever, WBC, Hb, N%, L%, NLR, M%, ESR, CRP, heliotrope rash, Gottron’s sign, V-neck sign, shawl sign, holster sign, Mechanic’s hands, Raynaud’s phenomenon, skin ulcers, proximal weakness, LDH, CK, ILD, Lung infection, arthritis/arthralgia
